# Economic injury levels for Asian citrus psyllid control in process oranges from mature trees with high incidence of huanglongbing

**DOI:** 10.1371/journal.pone.0175333

**Published:** 2017-04-20

**Authors:** Cesar Monzo, Philip A. Stansly

**Affiliations:** Entomology and Nematology Department, University of Florida/IFAS, Southwest Florida Research and Education Center, Immokalee, Florida, United States of America; Louisiana State University, UNITED STATES

## Abstract

The Asian citrus psyllid (ACP), *Diaphorina citri* Kuwayama, is the key pest of citrus wherever it occurs due to its role as vector of huanglongbing (HLB) also known as citrus greening disease. Insecticidal vector control is considered to be the primary strategy for HLB management and is typically intense owing to the severity of this disease. While this approach slows spread and also decreases severity of HLB once the disease is established, economic viability of increasingly frequent sprays is uncertain. Lacking until now were studies evaluating the optimum frequency of insecticide applications to mature trees during the growing season under conditions of high HLB incidence. We related different degrees of insecticide control with ACP abundance and ultimately, with HLB-associated yield losses in two four-year replicated experiments conducted in commercial groves of mature orange trees under high HLB incidence. Decisions on insecticide applications directed at ACP were made by project managers and confined to designated plots according to experimental design. All operational costs as well as production benefits were taken into account for economic analysis. The relationship between management costs, ACP abundance and HLB-associated economic losses based on current prices for process oranges was used to determine the optimum frequency and timing for insecticide applications during the growing season. Trees under the most intensive insecticidal control harbored fewest ACP resulting in greatest yields. The relationship between vector densities and yield loss was significant but differed between the two test orchards, possibly due to varying initial HLB infection levels, ACP populations or cultivar response. Based on these relationships, treatment thresholds during the growing season were obtained as a function of application costs, juice market prices and ACP densities. A conservative threshold for mature trees with high incidence of HLB would help maintain economic viability by reducing excessive insecticide sprays, thereby leaving more room for non-aggressive management tools such as biological control.

## Introduction

The Asian citrus psyllid (ACP), *Diaphorina citri* Kuwayama is a key pest in many citrus growing areas of the world due to its role as vector of huanglongbing (HLB) or citrus greening disease [1]. Except for the now rare *Candidatus* Liberibacter americanus, the causal agent of HLB in the Americas and Asia is thought to be *C*. L. asiaticus (CLAS), a phloem limited gram negative bacterium [2]. Huanglongbing reduces tree health, productivity and fruit quality [3]. Infected trees rapidly decline and become unproductive [4] unless corrective measures are taken [5, 6].

Huanglongbing is considered the most damaging of citrus diseases wherever present. Any citrus growing area where the disease and/or its vectors are detected must quickly adapt all production and management systems to avoid rapid collapse of the industry [7]. The medium-term consequences of HLB are well reflected in a study that evaluated economic impact to the Florida citrus industry during the first five years of coexistence with the disease [8]. These authors estimated 23% yield reductions from 2006 to 2011, revenue losses of $1.71 billion and the loss of 8,257 jobs direct or indirectly related to this industry. Downward production trend is still maintained in that area. In fact, NASS statistics show how orange production in Florida decreased from 6.94 to 3.33 billion tones (67.4% reduction) over the 8-year interval from the 2007–08 to the 2015–16 seasons [9].

Since no definitive curative remedies exist, the best strategy against HLB is clearly prevention. For earlier stages of the invasion, comprehensive monitoring programs for vector and HLB infected trees detection are recommended [10]. Under this scenario, intensive insecticidal vector control coupled with rogueing of symptomatic trees has been proposed for slowing disease spread [11, 12]. In the case of moderate to high HLB incidence such as currently found throughout the Florida citrus production area, infected-tree removal is no longer a viable option [7]. Nevertheless, recent studies have demonstrated that production under these conditions can be maintained, at least in the medium term, through insecticidal control of ACP and the foliar application of macro and micronutrients that otherwise cannot be normally acquired by roots owing to HLB-caused root dysfunction [5, 6, 13]. When the pathogen is not present, ACP is considered a minor pest that only causes significant damage at high densities [3]. When the insect vectors the disease, plant symptom severity and therefore tree decline progression is in part mediated by the number of infections per tree [12]. Recent studies proved the positive effects on yields of controlling ACP in commercial groves under high HLB incidence thus remarking the important role of bacterium re-inoculations on tree decline and CLAS titer [14].

Fear and severity of HLB have driven implementation of steadily intensifying insecticide programs in those citrus areas where the disease is present [15–17]. These programs pose environmental and public health concerns as well as side-effects to beneficial arthropod fauna of citrus agroecosystem [18]. Pesticide resistance derived from repeated use of similar modes of action is also a major concern for ACP management [19]. While insecticidal control of ACP has been demonstrated capable of significantly increasing production, the increased cost may not be justified if only moderately higher yields are obtained [5, 6, 20]. For this reason, it is important to seek optimum ACP management so that the cost of insecticide applications and other inputs do not exceed the expected value of the crop.

The use of broad-spectrum insecticides during tree dormancy to complement natural population decline at a time when collateral damage to beneficial fauna is minimized has proven to be an effective way of reducing ACP numbers well into the growing period [21]. Several ACP sampling techniques have been proposed and widely validated to monitor vector populations [22, 23]. Although citrus inhabiting natural enemies have not proven capable to alone reduce ACP numbers sufficiently, they contribute to mortality of this and other citrus pests and therefore merit inclusion in management programs through conservation measures together with elimination of unnecessary sprays and use of selective insecticides [18, 24, 25]. Biological control programs that include establishment and augmentation of the parasitoid *Tamarixia radiata* are currently being developed in Florida, California and elsewhere [26, 27]. Area-wide approaches promise to provide efficient ACP management in citrus growing areas where the vector is present [1]. Full use of this effort should include guidelines relating ACP populations to economic benefits of control.

Action thresholds based on pest densities are highly recommended for integrated pest management [28]. While an arbitrary threshold based on ACP adult densities obtained by stem-tap sampling was previously adopted for vector management [5, 6, 29] no prior studies were available to support this practice or provide economic thresholds for juice oranges under conditions of high HLB incidence. The objective of this study was therefore to provide a model for determining the level of ACP control that would optimize costs of ACP management in mature orange trees under conditions of high HLB incidence.

## Material and methods

### Study sites and experimental design

Two large-scale experiments were conducted in two commercial citrus groves in the southwest Florida citrus growing area (Hendry County, Florida, USA) from summer 2010 through spring 2014. The two experimental sites were located in large citrus groves and consisted of distinct blocks of orange trees individually managed in which owners and managers agreed to cede all pest management decisions to project managers. **Site 1**: A 10.3 ha block in a grove belonging to Bob Paul Inc. near LaBelle (26°41’04”N, 81°26’20”W), planted December 2001 with early season sweet oranges *Citrus sinensis* (L.) Obseck ‘Earlygold’, bud-grafted to ‘Carrizo’ citrange rootstock at a density of 231 trees ha^-1^. **Site 2:** A 5.4 ha block located in a large orchard belonging to Moreno Farms near Felda (26° 34' 0.6" N, 81° 26' 20.1"W) and planted in 1999 with late season sweet oranges *Citrus sinensis* (L.) Obseck ‘Valencia’ on ‘Swingle’ citrumelo rootstock at a density of 336 tree ha^-1^. Management followed conventional cultural practices [30] except for insecticidal control. In addition, a HLB foliar nutritional remediation program was applied throughout each block by the growers during major tree flushes (Table 1).

**Table 1:** Products, rates and costs of the remedial foliar nutrient applications program for HLB, and for canker management followed in Sites 1 and 2 between 2010 and 2014.

Product	Function	Company	Rate	$/unit	$/ha
13-0-44 fertilizer (KNO_3_)^1^	Macronutrient	Diamond R Fertilizer	9.53 kg/ha	1.64	15.62
Techmangan (MnS0_4_)^1^	Micronutrient	Diamond R Fertilizer	9.53 kg/ha	1.52	14.46
Zinc Sulfate (ZnSO_4_)^1^	Micronutrient	Diamond R Fertilizer	3.14 kg/ha	1.98	6.23
Sodium Molybdate (Na_2_MoO_4_)^1^	Micronutrient	Diamond R Fertilizer	0.06 kg/ha	52.83	3.15
Epsom Salts (MgSO_4_)^1^	Micronutrient	Diamond R Fertilizer	9.53 kg/ha	0.66	6.30
Beau-Ron ^®^ D (Na_2_B4O_7_·10H_2_O)^1^	Micronutrient	Drexel Chemical Co.	3.70 kg/ha	2.98	11.01
K-Phite^®^^1^	Macronutrient	Plant Food Systems	4.68 l/ha	7.40	34.60
Purespray Green^®^ (435 HMO)^1^	Adjuvant	Petro-Canada Lubricants, Inc.	28.06 l/ha	1.41	39.48
**Total cost of the program**^1^:					**130.83**
Kocide^®^ 3000 (Cu(OH)_2_)^2^	Canker management	DuPont ^™^	1.96 kg/ha	22.45	44.0
Cuprofix^®^ Ultra 40D^2^	Canker management	Cerexagri-Nisso LLC	2.24 kg/ha	35.71	80.6

^1^Remedial foliar nutrient program.

^2^Canker management.

The two study sites were divided into 16 plots (experimental units), each containing approximately 144 and 120 trees at sites 1 and 2 respectively. A randomized complete block design was used, each with four replications and 4 treatments: (1) ‘calendar’—monthly sprays of insecticides to control ACP, (2) ‘0.2 thsld’—insecticide applications based on a nominal threshold of 0.2 ACP adults per stem-tap sample plus two “dormant” (winter) sprays, (3) ‘0.7 thsld’—insecticide applications based on nominal threshold of 0.7 ACP adults per stem-tap sample plus one dormant spray, and (4) ‘no insecticide’—a control treatment where no insecticide applications to control ACP. The ‘calendar’ treatment was aimed to keep ACP densities as low as possible by following an intensive spray schedule using a rotation of insecticides. At the other extreme was the ‘no insecticide’ control treatment where ACP populations were allowed to vary without insecticide intervention. The two nominal threshold treatments were set to create intermediate ACP densities and control costs.

### Pest and disease management

All Insecticide treatments were made in designated plots with an Air-O-Fan airblast sprayer equipped with Albuz^®^ ATR hollow cone nozzles providing an 80° spray pattern with five nozzles, two of 2.5 and three of 4 L/min, operating at 300 PSI and 3 km/h delivering a total of 887 L/ha. Monthly applications against ACP were initiated in 2010 in plots designated for ‘calendar’ treatment of both study sites (Tables 2 and 3). Broad-spectrum products (organophosphates, carbamates and pyrethroids) were generally restricted to the winter and the end of the summer whereas more selective insecticides were chosen preferentially during the growing season to rotate modes of action, control other pest present and reduce impacts on beneficial arthropod fauna [17–19, 21, 31]. Ten modes of action were rotated to avoid inducing resistance in *D*. *citri*. Thus, insecticide choices during the growing season were made on the basis of efficacy, timeliness, and resistance management rather than product cost. Occasional sprays required for control of other pests or diseases were chosen for minimal impact on ACP and made over the entire study site to avoid confounding treatment effects.

**Table 2:** Pest and disease chemical foliar applications and their cost ($/ha) for the four ACP control treatments evaluated: no insecticide (1), calendar applications (2), 0.2 threshold (3) and 0.7 threshold (4), from summer 2010 to fall 2013 in the ‘Earlygold’ planted block (Site 1). ‘Nutrients’ refers to the foliar nutritional remediation spray program used by the growers.

Dates	Active Ingredient (Brand Name)	Target	Rate^1^	435 HMO^2^ (vol %)	Treatment Sprayed	Insecticide Cost ($/ha)	435 HMO^2^ Cost ($/ha)	Application Cost ($/ha)	Total Cost ($/ha)
30-Jul-10	Spinetoram (Delegate^®^WG)	ACP	0.315 kg/ha	2	2,3	60.2	32.9	59.6	**152.7**
13-Oct-10	Dimethoate (Dimethoate^®^ 4E)	ACP	1.121 kg/ha	0	2	13.6	0.0	59.6	**73.1**
20-Jan-11	Fenpropathrin (Danitol^®^ 2.4 EC)^3^	ACP	0.585 l/ha	0	2,3,4	24.7	0.0	59.6	**84.3**
4-Mar-11	Nutrients + Copper (Kocide^®^ 3000)	HLB + Canker	1	***	1,2,3,4	176.7	0.0	59.6	**236.2**
16-Mar-11	Diflubenzuron (Micromite^®^ 80WGS)	ACP	0.438 kg/ha	2	2	61.5	32.9	59.6	**153.9**
15-Apr-11	Carbaryl (Sevin XLR^®^Plus)	ACP	7.015 l/ha	0	2	55.6	0.0	59.6	**115.2**
18-May-11	Spinetoram (Delegate^®^WG)	ACP	0.315 kg/ha	2	2	60.2	32.9	59.6	**152.7**
15-Jun-11	Imidacloprid (Admire Pro^®^)	ACP	0.315 kg/ha	2	2	44.4	32.9	59.6	**136.9**
15-Jul-11	Nutrients	HLB	1	***	1,2,3,4	138.1	0.0	59.6	**197.7**
28-Jul-11	Abamectine (Agri-Mek^®^ SC)	ACP	0.256 l/ha	2	2	4.4	32.9	59.6	**96.9**
1-Aug-11	Sulfur WP (Microthiol Disperss^®^)	Rust mite	16.81 kg/ha		1,2,3,4	33.4	0.0	59.6	**92.9**
9-Aug-11	Nutrients + Copper (Cuprofix^®^ Ultra 40D)	HLB + Canker	1	***	1,2,3,4	170.7	0.0	59.6	**230.3**
19-Aug-11	Malathion (Gowan^®^ Malathion 8F)	ACP	2.923 l/ha	0	2	38.5	0.0	59.6	**98.1**
16-Sep-11	Fenpropathrin (Danitol^®^ 2.4 EC)	ACP	0.585 l/ha	0	2	24.7	0.0	59.6	**84.3**
30-Sep-11	Nutrients + Copper (Cuprofix^®^ Ultra 40D)	HLB + Canker	1	***	1,2,3,4	170.7	0.0	59.6	**230.3**
2-Nov-11	Spirotetramat (Movento MPC^®^)	ACP	1.169 l/ha	2	2	62.2	32.9	59.6	**154.7**
2-Dec-11	Carbaryl (Sevin XLR^®^Plus)	ACP	7.015 l/ha	0	2	55.6	0.0	59.6	**115.2**
19-Dec-11	Phosmet (Imidan^®^ 70-W)^3^	ACP	1.121 kg/ha	0	2,3	23.5	0.0	59.6	**83.0**
12-Jan-12	Zeta-cypermethrin (Mustang^®^)^4^	ACP	0.301 kg/ha	0	2,3,4	11.4	0.0	59.6	**70.9**
29-Feb-12	Nutrients	HLB	1	***	1,2,3,4	138.1	0.0	59.6	**197.7**
7-Mar-12	Spirotetramat (Movento MPC^®^)	ACP	1.169 l/ha	2	2	62.2	32.9	59.6	**154.7**
26-Apr-12	Diflubenzuron (Micromite^®^ 80WGS)	ACP	0.438 kg/ha	2	2	61.5	32.9	59.6	**153.9**
1-May-12	Nutrients + Copper (Kocide^®^ 3000)	HLB + Canker	1	***	1,2,3,4	176.7	0.0	59.6	**236.2**
6-Jun-12	Abamectin (Agri-Mek^®^ SC) + Nutrients	HLB +Rust mite	0.256 l/ha		1,2,3,4	159.9	0.0	59.6	**219.4**
29-Jun-12	Spinetoram (Delegate^®^WG)	ACP	0.315 kg/ha	2	2	60.2	32.9	59.6	**152.7**
23-Jul-12	Nutrients + Copper (Cuprofix^®^ Ultra 40D)	HLB + Canker	1	***	1,2,3,4	170.7	0.0	59.6	**230.3**
10-Aug-12	Imidacloprid (Admire Pro^®^)	ACP	0.315 kg/ha	2	2	44.4	32.9	59.6	**136.9**
13-Aug-12	Nutrients	HLB	1	***	1,2,3,4	138.1	0.0	59.6	**197.7**
18-Sep-12	Dimethoate (Dimethoate^®^ 4E)	ACP	1.121 kg/ha	0	2	13.6	0.0	59.6	**73.1**
1-Oct-12	Sulfur WP (Microthiol Disperss^®^)	Rust mite	16.81 kg/ha	0	1,2,3,4	33.4	0.0	59.6	**92.9**
19-Oct-12	Fenpyroximate (Portal^®^)	ACP	4.677 l/ha	0	2	37.6	0.0	59.6	**97.1**
17-Dec-12	Zeta-cypermethrin (Mustang^®^)^3^	ACP	0.301 kg/ha	0	2,3	11.4	0.0	59.6	**70.9**
11-Jan-13	Phosmet (Imidan^®^ 70-W)^4^	ACP	1.121 kg/ha	0	2,3,4	23.5	0.0	59.6	**83.0**
28-Feb-13	Nutrients	HLB	1	***	1,2,3,4	138.1	0.0	59.6	**197.7**
11-Mar-13	Spirotetramat (Movento MPC^®^)	ACP	1.169 l/ha	2	2	62.2	32.9	59.6	**154.7**
29-Apr-13	Diflubenzuron (Micromite^®^ 80WGS)	ACP	0.438 kg/ha	2	2,3	61.5	32.9	59.6	**153.9**
11-Jun-13	Spinetoram (Delegate^®^WG)	ACP	0.315 kg/ha	2	2,3	60.2	32.9	59.6	**152.7**
24-Jul-13	Imidacloprid (Admire Pro^®^)	ACP	0.315 kg/ha	2	2,3	44.4	32.9	59.6	**136.9**
25-Jul-13	Nutrients + Copper (Kocide^®^ 3000)	HLB + Canker	1	***	1,2,3,4	176.7	0.0	59.6	**236.2**
29-Aug-13	Clorpyrifos (Lorsban^®^ Advanced)	ACP	5.846 l/ha	0	2	59.1	0.0	59.6	**118.6**
24-Sep-13	Sulfoxaflor (Closer SC^®^)	ACP	0.365 l/ha	2	2	77.4	32.9	59.6	**169.8**
21-Oct-13	Nutrients	HLB	1	***	1,2,3,4	78.6	0.0	59.6	**138.1**
28-Oct-13	Carbaryl (Sevin XLR^®^Plus)	ACP	7.015 l/ha	0	2	55.6	0.0	59.6	**115.2**

^1^Rates on foliar nutritional applications equal to 1 refer to products rates presented on Table 1

^2^435 HMO: 435 Horticultural mineral oil.

^3^First dormant spray of the season.

^4^Second dormant spray of the season.

**Table 3:** Pest and disease chemical foliar applications and their cost ($/ha) for the four ACP control treatments evaluated: no insecticide (1), calendar applications (2), 0.2 threshold (3) and 0.7 threshold (4), from summer 2010 to spring 2014 in the ‘Valencia’ planted block (Site 2). ‘Nutrients’ is refers to the followed foliar nutritional remediation spray program used by the grower.

Dates	Active Ingredient (Brand Name)	Target	Rate^1^	435 HMO^2^ (vol %)	Treatment Sprayed	Insecticide Cost ($/ha)	435 HMO^2^ Cost ($/ha)	Application Cost ($/ha)	Total Cost ($/ha)
2-Sep-10	Nutrients + Copper (Cuprofix^®^ Ultra 40D)	HLB + Canker	1	***	1,2,3,4	170.7	0.0	59.6	**230.3**
22-Sep-10	Dimethoate (Dimethoate^®^ 4E)	ACP	1.121 kg/ha	0	2	13.6	0.0	59.6	**73.1**
21-Jan-11	Fenpropathrin (Danitol^®^ 2.4 EC)^3^	ACP	0.585 l/ha	0	2,3,4	24.7	0.0	59.6	**84.3**
14-Mar-11	Nutrients + Copper (Kocide^®^ 3000)	HLB + Canker	1	***	1,2,3,4	176.7	0.0	59.6	**236.2**
16-Mar-11	Diflubenzuron (Micromite^®^ 80WGS)	ACP	0.438 kg/ha	2	2	61.5	32.9	59.6	**153.9**
18-Apr-11	Carbaryl (Sevin XLR^®^Plus)	ACP	7.015 l/ha	0	2	55.6	0.0	59.6	**115.2**
16-May-11	Spinetoram (Delegate^®^WG)	ACP	0.315 kg/ha	2	2	60.2	32.9	59.6	**152.7**
17-Jun-11	Imidacloprid (Admire Pro^®^)	ACP	0.315 kg/ha	2	2	44.4	32.9	59.6	**136.9**
14-Jul-11	Abamectine (Agri-Mek^®^ SC)	ACP	0.256 l/ha	2	2	4.4	32.9	59.6	**96.9**
5-Aug-11	Nutrients	HLB	1	***	1,2,3,4	138.1	0.0	59.6	**197.7**
15-Aug-11	Malathion (Gowan^®^ Malathion 8F)	ACP	2.923 l/ha	0	2	38.5	0.0	59.6	**98.1**
15-Sep-11	Fenpropathrin (Danitol^®^ 2.4 EC)	ACP	0.585 l/ha	0	2	24.7	0.0	59.6	**84.3**
20-Oct-11	Spirotetramat (Movento MPC^®^)	ACP	1.169 l/ha	2	2	62.2	32.9	59.6	**154.7**
26-Oct-11	Nutrients	HLB	1	***	1,2,3,4	138.1	0.0	59.6	**197.7**
7-Dec-11	Carbaryl (Sevin XLR^®^Plus)	ACP	7.015 l/ha	0	2	55.6	0.0	59.6	**115.2**
20-Dec-11	Phosmet (Imidan^®^ 70-W)^3^	ACP	1.121 kg/ha	0	2,3	23.5	0.0	59.6	**83.0**
19-Jan-12	Zeta-cypermethrin (Mustang^®^)^4^	ACP	0.301 kg/ha	0	2,3,4	11.4	0.0	59.6	**70.9**
1-Mar-12	Nutrients (Half the rate)	HLB	1/2	***	1,2,3,4	68.9	0.0	59.6	**128.5**
23-Mar-12	Clorpyrifos (Lorsban^®^ 4EC)	Rust mite	7.015 l/ha		1,2,3,4	59.1	0.0	59.6	**118.6**
23-Mar-12	Nutrients (Half the rate)	HLB	1/2	***	1,2,3,4	68.9	0.0	59.6	**128.5**
16-Apr-12	Diflubenzuron (Micromite^®^ 80WGS)	ACP	0.438 kg/ha	2	2	61.5	32.9	59.6	**153.9**
11-May-12	Nutrients (half the rate)	HLB	1/2	***	1,2,3,4	9.6	0.0	59.6	**69.2**
24-May-12	Spinetoram (Delegate^®^WG)	ACP	0.315 kg/ha	2	2	60.2	32.9	59.6	**152.7**
15-Jun-12	Nutrients (half the rate)	HLB	1/2	***	1,2,3,4	68.9	0.0	59.6	**128.5**
22-Jun-12	Abamectine (Agri-Mek^®^ SC)	ACP	0.256 l/ha	2	2	4.4	32.9	59.6	**96.9**
26-Jul-12	Nutrients + Copper (Kocide^®^ 3000)	HLB + Canker	1	***	1,2,3,4	176.7	0.0	59.6	**236.2**
3-Aug-12	Imidacloprid (Admire Pro^®^)	ACP	0.315 kg/ha	2	2	44.4	32.9	59.6	**136.9**
15-Aug-12	Nutrients	HLB	1	***	1,2,3,4	78.6	0.0	59.6	**138.1**
30-Aug-12	Dimethoate (Dimethoate^®^ 4E)	ACP	1.121 kg/ha	0	2,3	13.6	0.0	59.6	**73.1**
12-Oct-12	Fenpyroximate (Portal^®^)	ACP	4.677 l/ha	0	2	37.6	0.0	59.6	**97.1**
14-Dec-12	Zeta-cypermethrin (Mustang^®^)^3^	ACP	0.301 kg/ha	0	2,3	11.4	0.0	59.6	**70.9**
11-Jan-13	Phosmet (Imidan^®^ 70-W)^4^	ACP	1.121 kg/ha	0	2,3,4	23.5	0.0	59.6	**83.0**
8-Apr-13	Fenpropathrin (Danitol^®^ 2.4 EC)	ACP	0.585 l/ha	0	2,3,4	24.7	0.0	59.6	**84.3**
8-Apr-13	Nutrients	HLB	1	***	1,2,3,4	78.6	0.0	59.6	**138.1**
29-Apr-13	Nutrients (half the rate)	HLB	1/2	***	1,2,3,4	68.9	0.0	59.6	**128.5**
6-May-13	Diflubenzuron (Micromite^®^ 80WGS)	ACP	0.438 kg/ha	2	2	61.5	32.9	59.6	**153.9**
11-Jun-13	Spinetoram (Delegate^®^WG)	ACP	0.315 kg/ha	2	2,3	60.2	32.9	59.6	**152.7**
24-Jul-13	Imidacloprid (Admire Pro^®^)	ACP	0.315 kg/ha	2	2	44.4	32.9	59.6	**136.9**
25-Jul-13	Nutrients + Copper (Kocide^®^ 3000)	HLB + Canker	1	***	1,2,3,4	176.7	0.0	59.6	**236.2**
29-Aug-13	Clorpyrifos (Lorsban^®^ Advanced)	ACP	5.846 l/ha	0	2	59.1	0.0	59.6	**118.6**
24-Sep-13	Sulfoxaflor (Closer SC^®^)	ACP	0.365 l/ha	2	2	77.4	32.9	59.6	**169.8**
28-Oct-13	Carbaryl (Sevin XLR^®^Plus)	ACP	7.015 l/ha	0	2	55.6	0.0	59.6	**115.2**
17-Dec-13	Phosmet (Imidan^®^ 70-W)^3^	ACP	1.121 kg/ha	0	2,3	23.5	0.0	59.6	**83.0**
22-Jan-14	Fenpropathrin (Danitol^®^ 2.4 EC)^4^	ACP	0.585 l/ha	0	2,3,4	24.7	0.0	59.6	**0.0**

^1^Rates on foliar nutritional applications equal to 1 refer to products rates presented on Table 1, rates equal to ½ indicate that half the regular rate was used in that particular spray.

^2^435 HMO: 435 Horticultural mineral oil.

^3^First dormant spray of the season.

^4^Second dormant spray of the season.

Sprays for the two additional treatments (‘0.2 thsld’ and ‘0.7 thsld’) were triggered during the growing season if and when tap samples averaged over all 4 replicates reached or exceeded nominal thresholds. At that time, all 4 replicates of the treatment were sprayed using the same product chosen for the ‘calendar’ treatment that month. Dormant season insecticide applications in these two treatments were made without consideration of thresholds in conformance with synchronized dormant sprays organized in the local “citrus health management area” [CHMA www.flchma.org]. The ‘0.7 thsld’ treatment received its dormant spray in January whereas ‘0.2 thsld’ treatment received two dormant sprays, one in December and one in January. Each site was sprayed at least once per season with copper-based products to control citrus canker, *Xanthomonas axonopodis* pv. *citri*. Site 1 was sprayed by the grower in August 2011 and October 2012 with sulfur (Microthiol Disperss^®^) to control citrus rustmite, *Phyllocoptruta oleivora* (Ashmead) (Acari: Eriophyidae). Clorpyrifos (Lorsban^®^ 4EC) was applied inadvertently by the grower over all of Site 2 for general pest control in March 2012.

### Asian citrus psyllid monitoring

Density of ACP adults was monitored every other week from 7 July 2010 through 6 November 2013 at Site 1 and from 12 August 2010 to 24 February 2014 at Site 2 by stem-tap on two sides of 10 randomly selected trees in the vicinity of two previously selected stops per plot (320 trees sampled per grove and date) [23]. A 22 × 28 cm plastic laminated white paper sheet was held horizontally about 30 cm underneath a randomly chosen branch, which was then struck sharply three times with a length of PVC pipe. ACP adults falling on the sheet were quickly counted and the two numbers averaged to obtain the sample unit: “ACP adults per stem-tap and tree” [23]. Biweekly mean numbers of ACP adults per stem-tap were used to determine whether insecticide applications were necessary in plots designated for the ‘0.2 thsld’ and ‘0.7 thsld’ treatments. ACP adult cumulative numbers per stem-tap and tree (κ) were summed over the season through each harvest date in fall for ‘Earlygold’ (Site 1) and spring for ‘Valencia’ (Site 2).

### HLB incidence

The proportion of trees testing positive for *Candidatus* Liberibacter asiaticus in each treatment was evaluated twice per year, in midsummer and at the end of winter, beginning in summer 2010 before treatments were initiated, through summer 2013. Samples consisted of 20 randomly collected leaves per tree, five at each cardinal point, from 20 randomly sampled trees per plot. Samples were taken to the laboratory and DNA extracted from a 50 mg dry weight subsample of petiole tissue using the Promega Wizard ^®^ 96 DNA Plant isolation kit (Promega, USA). Real-time qPCR was conducted with an ABI 7500 Fast Real-Time PCR System (Applied Biosystems, Foster City, CA) in a 20 μl volume using HLBas/HLBr and HLBp *Candidatus* Liberibacter asiaticus primers [32]. The standard amplification protocol was initial denaturation at 95°C followed by 40 cycles of reactions (95°C for 3 s, 60°C for 30 s). Data was analyzed using Applied Biosystems 7500 system SDS software. Samples were considered positive for Ct values less or equal to 36 [33].

### Harvest evaluations

All marketable fruit was harvested by plot in December 7–13 in 2010 (2010–11 season), November 16–20 in 2011 (2011–12 season), November 14–23 in 2012 (2012–13 season) and November 18–21 in 2013 (2013–14 season) in Site 1, and January 28 to February 3 in 2011 (2010–11 season), January 23 to February 3 in 2012 (2011–12 season), in February 24–26 in 2013 (2012–13 season) and March 6–12 2014 (2013–14 season) in Site 2. The mass of fruit from each plot (kg of fruit per ha) was estimated by counting full and partially full tubs during the harvest. Tubs were 784.1 dm^3^ in volume and designed to contain a volume of ten standard boxes of oranges (40.8 kg per box) [34] when totally full. Partially full tubs were measured from the top of the container to the level of harvested fruit and the corresponding fruit volume and weight was calculated.

Juice quality, measured as ‘kg of juice per kg of fruit’, ‘kg of soluble solids per kg of fruit’, acidity, ‘total degrees brix’ and ‘brix/acid’ ratio, was evaluated from a 2 bushel composite random fruit sample collected in each plot one week prior to initiating harvest. Samples were brought to the University of Florida citrus quality laboratory in Lake Alfred, FL where juice was extracted and de-aerated under vacuum for 2–3 minutes. Soluble solids content was measured by hydrometer and titratable acidity as citric acid, pH endpoint 8.2. Since production destined for the juice industry and processed oranges prices are valued by the amount of soluble solids, yields (kg of solids per ha) were calculated by multiplying the harvested kg of fruit per ha from each plot by estimated solids (kg of soluble solids per kg of fruit) obtained from the corresponding treatment through juice analyses. When no significant treatment effect was found, the average value for the entire grove was used for calculations.

### Economic study

The economic viability of each tested treatment was studied using yield data from all harvests. Incomes for processed oranges ($/ha) were estimated for the different treatments in each year by comparing changes in revenues under a range of fruit prices representing those obtained for early and late-season juice oranges in Florida during the last three seasons [35].

Production costs were divided into the following grove operations [36]: pest management, foliar nutrition program, canker management, fruit picking, hauling and Florida Department of Citrus assessment costs, ACP monitoring, weed management, ground fertilizer, pruning practices, irrigation and tree replacement costs (Table 4). Property and district taxes, management fees charged by professional caretakers, interest charges on operating capital and interest on investment capital were also included as indirect production costs. Foliar chemical application costs for pest and disease management were separated into material and application costs (Tables 2 and 3). Product prices were provided by chemical and fertilizer vendors. Application costs ($59 per ha) were those estimated by Roka *et al*. [36] for 935 L per ha (100 gallon per acre) air-blast chemical applications in Central and Southwest Florida. Similarly, we used the same estimate as these authors for all harvesting costs (Pick and roadside: $0.063 per kg of harvested fruit; haul: $0.018 per kg of fruit; FDOC assessment: $0.006 per kg of fruit). ACP scouting costs were estimated taking into account the time spent on monitoring each plot and were only applied to those treatments where the decision to spray was based on nominal thresholds (0.2 and 0.7 thsld). For the remaining operations, we used costs estimated by Roka *et al*. [36] for orange juice production in southwest Florida.

**Table 4:** Estimated production costs ($/ha) for juice oranges in the two sites where the experiments were conducted: 2010–11, 2011–12, 2012–13 and 2013–14 seasons.

			Insecticide management^2^	Foliar nutritional management	Canker management	Picking and hauling costs	ACP monitoring	Weed management	Ground fertilizer	Pruning	Irrigation	Tree replacement	District taxes	Total ($/ha)
**Site 1**	**2010–11**^1^	**No insecticide**	1643.3	0.0	0.0	1886	0	504	1013	37	534	566	371	**6554**
		**0.7 thsld**	1643.3	0.0	0.0	1855	0	504	1013	37	534	566	371	**6523**
		**0.2 thsld**	1643.3	0.0	0.0	1731	0	504	1013	37	534	566	371	**6399**
		**Calendar**	1643.3	0.0	0.0	1914	0	504	1013	37	534	566	371	**6582**
	**2011–12**	**No insecticide**	93.0 (n = 1)	761.3	205.1	1832	0	974	1240	37	667	680	371	**6860**
		**0.7 thsld**	177.3 (n = 2)	761.3	205.1	1948	141	974	1240	37	667	680	371	**7201**
		**0.2 thsld**	177.3 (n = 2)	761.3	205.1	1871	141	974	1240	37	667	680	371	**7124**
		**Calendar**	1170.3 (n = 10)	761.3	205.1	2322	0	974	1240	37	667	680	371	**8428**
	**2012–13**	**No insecticide**	93.0 (n = 1)	820.9	124.5	2104	0	974	1240	37	667	680	371	**7111**
		**0.7 thsld**	163.9 (n = 3)	820.9	124.5	2179	141	974	1240	37	667	680	371	**7398**
		**0.2 thsld**	247.0 (n = 3)	820.9	124.5	2004	141	974	1240	37	667	680	371	**7306**
		**Calendar**	1195.0 (n = 11)	820.9	124.5	2330	0	974	1240	37	667	680	371	**8439**
	**2013–14**	**No insecticide**	0.0 (n = 0)	571.0	44.0	2052	0	974	1240	37	667	680	371	**6636**
		**0.7 thsld**	83.1 (n = 1)	571.0	44.0	1941	141	974	1240	37	667	680	371	**6749**
		**0.2 thsld**	597.7 (n = 5)	571.0	44.0	2136	141	974	1240	37	667	680	371	**7458**
		**Calendar**	1156.2 (n = 9)	571.0	44.0	2418	0	974	1240	37	667	680	371	**8158**
**Site 2**	**2010–11**^1^	**No insecticide**	1643.3	0.0	0.0	2583	0	504	1013	37	534	566	371	**7251**
		**0.7 thsld**	1643.3	0.0	0.0	2294	0	504	1013	37	534	566	371	**6962**
		**0.2 thsld**	1643.3	0.0	0.0	1880	0	504	1013	37	534	566	371	**6548**
		**Calendar**	1643.3	0.0	0.0	2431	0	504	1013	37	534	566	371	**7099**
	**2011–12**	**No insecticide**	0 (n = 0)	571	44	2058	0	974	1240	37	667	680	371	**6641**
		**0.7 thsld**	71 (n = 1)	571	44	2108	141	974	1240	37	667	680	371	**6904**
		**0.2 thsld**	154 (n = 2)	571	44	2308	141	974	1240	37	667	680	371	**7186**
		**Calendar**	1262 (n = 11)	571	44	2411	0	974	1240	37	667	680	371	**8257**
	**2012–13**	**No insecticide**	83 (n = 1)	880	44	2152	0	974	1240	37	667	680	371	**7128**
		**0.7 thsld**	202 (n = 2)	880	44	2012	141	974	1240	37	667	680	371	**7248**
		**0.2 thsld**	346 (n = 4)	880	44	2442	141	974	1240	37	667	680	371	**7822**
		**Calendar**	1043 (n = 10)	880	44	2796	0	974	1240	37	667	680	371	**8733**
	**2013–14**	**No insecticide**	0 (n = 0)	506	44	1506	0	974	1240	37	667	680	371	**6024**
		**0.7 thsld**	169 (n = 2)	506	44	1794	141	974	1240	37	667	680	371	**6622**
		**0.2 thsld**	404 (n = 4)	506	44	2182	141	974	1240	37	667	680	371	**7245**
		**Calendar**	1099 (n = 9)	506	44	2305	0	974	1240	37	667	680	371	**7922**

^1^ Management costs during the first season were considered as the average estimated for southwest Florida by Muraro and Roka (2013).

^2^ The number within brackets indicates the number of insecticide applications per season.

Based on total management costs and yields, a break-even price ($/kg of solids) to recover production costs was calculated for each site, treatment and season and for the last three seasons together. Profitability ($/ha) was also calculated by subtracting total management costs from income obtained at the different price scenarios evaluated.

### Statistical analyses

Differences in ACP adult cumulative numbers among treatments through harvest for each year and site were assessed using repeated measures analysis based on generalized mixed models. Data was assumed to be Poisson distributed; ‘block’ (replicate) was considered as a random factor, ‘treatment’ as fixed factor and ‘time’ as the repeated fixed effect. Several covariance structures were tested and an autoregressive structure was selected based on Akaikei and Bayesian information criteria (AIC and BIC respectively). Treatment effects on the proportion of trees testing positive for HLB by qPCR for each sample date were evaluated for Site 2 by generalized mixed model analysis where data was assumed to be binomially distributed, ‘block’ was considered as a random factor and ‘treatment’ as a fixed factor. Treatment effects on juice quality parameters were assessed using general mixed model analyses where ‘block’ was considered as a random factor and ‘treatment’ as a fixed factor. Changes through time in yields and differences in this parameter among treatments were analyzed for each Site through repeated measures analysis using generalized mixed models. Data from Site 1 was found to be normally distributed whereas data from Site 2 were slightly overdispersed and a Poisson distribution assumption for data resulted in better fit based in the scaled Pearson statistic and Akaikei and Bayesian information criteria. ‘Block’ was considered as a random factor, ‘treatment’ as fixed factor and ‘time’ as the repeated fixed effect. Autoregressive and heterogeneous compound symmetric covariance structures were selected for Sites 1 and 2 respectively based on AIC and BIC. Post-hoc t-test (LSD) comparisons were made in case of any significant effect (*P* < 0.05) for all analyses.

### Economic injury level model

A model was developed for each site and for pooled data from both sites to predict yield losses caused by the pest in 10–15 year-old blocks of orange trees under moderate to high HLB incidence. The model used 2013–14 harvest results to forecast a target number of psyllids per season that would balance costs of a given insecticidal ACP management program. Economic injury level was calculated as the vector density at which additional costs for ACP management were equal to the estimated losses caused by this vector-disease system [28] using Eq (1).

$$C=P\cdot Y_{m}\cdot \rho$$ (1)

Where C in $/ha represents ACP management costs, *P* is the harvest price in $/kg of solids and *ρ* is the effect on the ACP population on maximum yield Y_m_ when the pest is not present or at its lowest practical level. In our study, *Y*_*m*_ was estimated based on the average yield from the last harvest (2013–14) in the ‘calendar’ plots expressed as kg of solids per ha, where ACP numbers were as close to 0 as could reasonably be expected. Proportional yield losses for any of the other 12 plots (*i*) of each grove (*ρ*) were calculated with respect to the average yield for the ‘calendar’ treatment as:

$$\rho =\frac{Y_{m-Y_{i}}}{Y_{m}}$$ (2)

The variable *ρ* represented yield loss without indicating any particular cause. Non-linear least-squares regression analysis was used to explain the relationship between yield losses and ACP cumulative number per tree and season (κ) for each plot from the beginning of the experiments until the 2013–14 harvest. Data was fitted to a rectangular hyperbolic Eq (3) [28, 37–39] by using the Newton-Raphson iterative estimation procedure that included two parameters: ‘I’ and ‘A’:

$$\rho =\;\frac{I\cdot \kappa }{1+\frac{I\cdot \kappa }{A}}\cdot 100^{-1}$$ (3)

Where ‘I’ was defined as the horizontal asymptote of the function, which in our study represented the maximum yield loss (%) that the pest could induce after four seasons. ‘A’, was the slope of the curve at the origin which represented the rate of yield loss at low pest density. Results from two plots in each site were considered outliers and therefore removed from the equation.

Once *ρ* was expressed as a function of vector densities, Eq (1) was written as:

$$C=P\cdot Y_{m}\cdot \left(\frac{I\cdot k}{1+\frac{I\cdot k}{A}}\right)\cdot 100^{-1}$$ (4)

Given known values for *C*, *P* and *Y*_*m*_, the vector density, expressed as ACP adult cumulative number per tree and season (*κ*) that balanced the two sides of Eq (4) would define the EIL for these conditions.

### EIL implementation

An insecticide vector control approach was adopted, based on yield losses and ACP densities relationships, and taking into account the variable nature of management costs and juice market prices. The decision to spray during the growing season in mature citrus groves under high HLB incidence would be made according to ACP data collected during that season through stem-tap sampling. The program would commence with two dormant-season spray applications during winter, irrespective to ACP densities [21, 40]. Meanwhile, a running estimate would be maintained charting the number of ACP adults over the season and expressed as ACP cumulative number per stem. It would be time to spray again when the sum of costs of the two dormant-season sprays, a first insecticide application during the growing season and scouting equaled estimated losses based on ACP numbers. A new threshold would then be calculated to balance the costs listed above plus the additional application. The procedure would be repeated until the end of the growing season.

## Results

### Asian citrus psyllid monitoring

**Fig 1:**
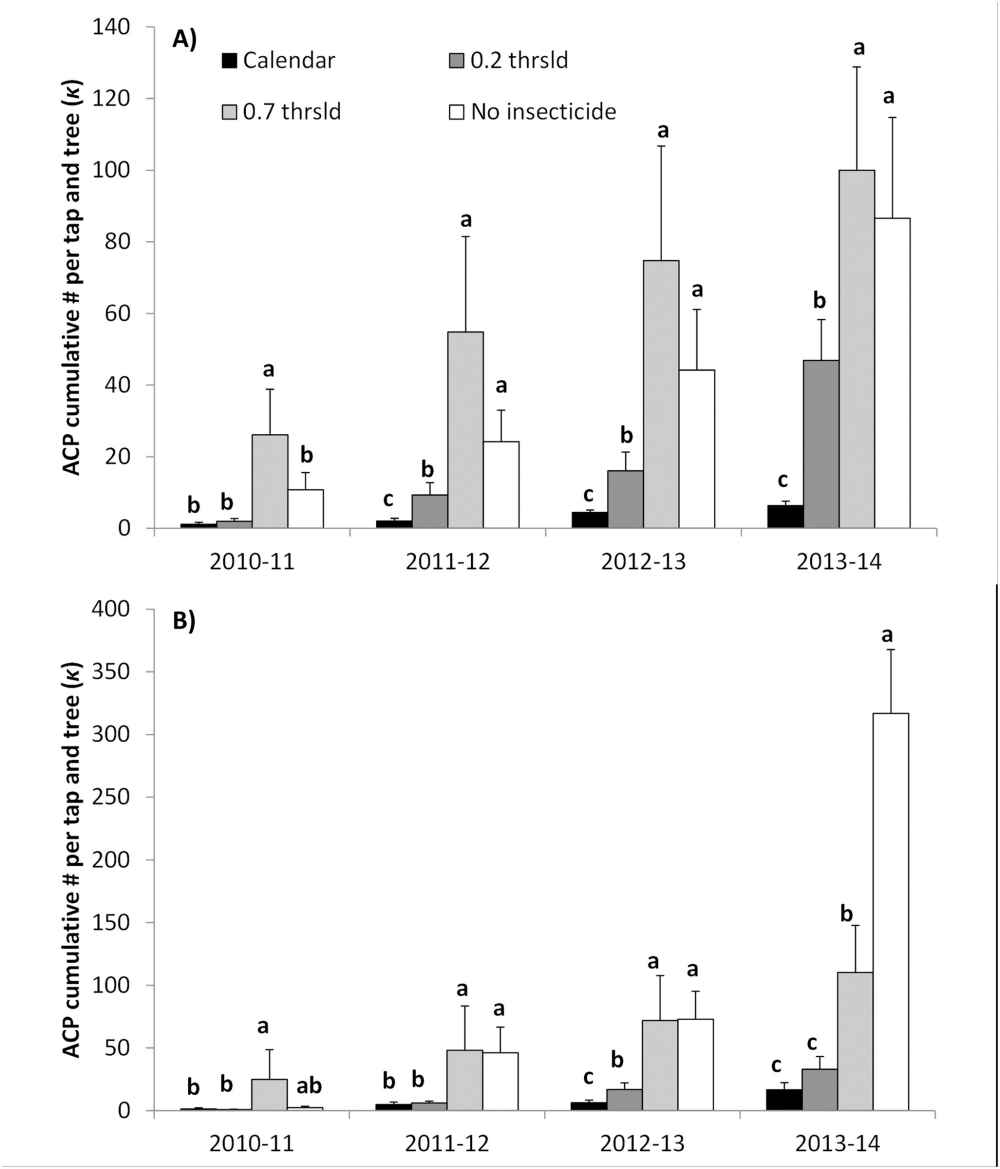
ACP adult cumulative number (κ) per stem-tap and tree (mean ± SE). Data obtained in plots under four different vector management strategies: (1) monthly calendar insecticide applications ‘calendar’, (2) insecticide applications based on a nominal threshold of 0.2 adults per stem-tap ‘0.2 thsld’, (3) applications based on a nominal threshold of 0.7 adults per stem-tap ‘0.7 thsld’, and (4) no insecticide applications to control ACP ‘no insecticide’ in Site 1 **A)**, and Site 2 **B)**. Cumulative numbers where calculated from the beginning of the experiments to the date of each harvest. For each harvest, different letters indicate significant differences among treatments (LSD Means: P <0.05).

Insecticide management significantly reduced ACP adult abundance at both sites (treatment effect: F = 17.88; df = 3, 69; *P* < 0.0001 for Site 1, and F = 12.71; df = 3, 27.9; *P* < 0.0001 for Site 2). Lowest ACP cumulative values were seen at both sites throughout the study in plots receiving calendar insecticide applications against the psyllid compared to the untreated control or the ‘0.7 thsld’ treatment (Fig 1). A significant interaction effect between treatment and time at Site 2 (F = 3.57; df = 9, 102.8; *P* = 0.0007) reflected the fact higher ACP numbers were seen early on in ‘0.7 thsld’ treatment plots compared to ‘no insecticide’ treatment plots, although the situation reversed toward the end of the study. This effect was nevertheless not seen at Site 1 (F = 1.17; df = 9, 69; *P* = 0.3282).

### HLB incidence

**Fig 2:**
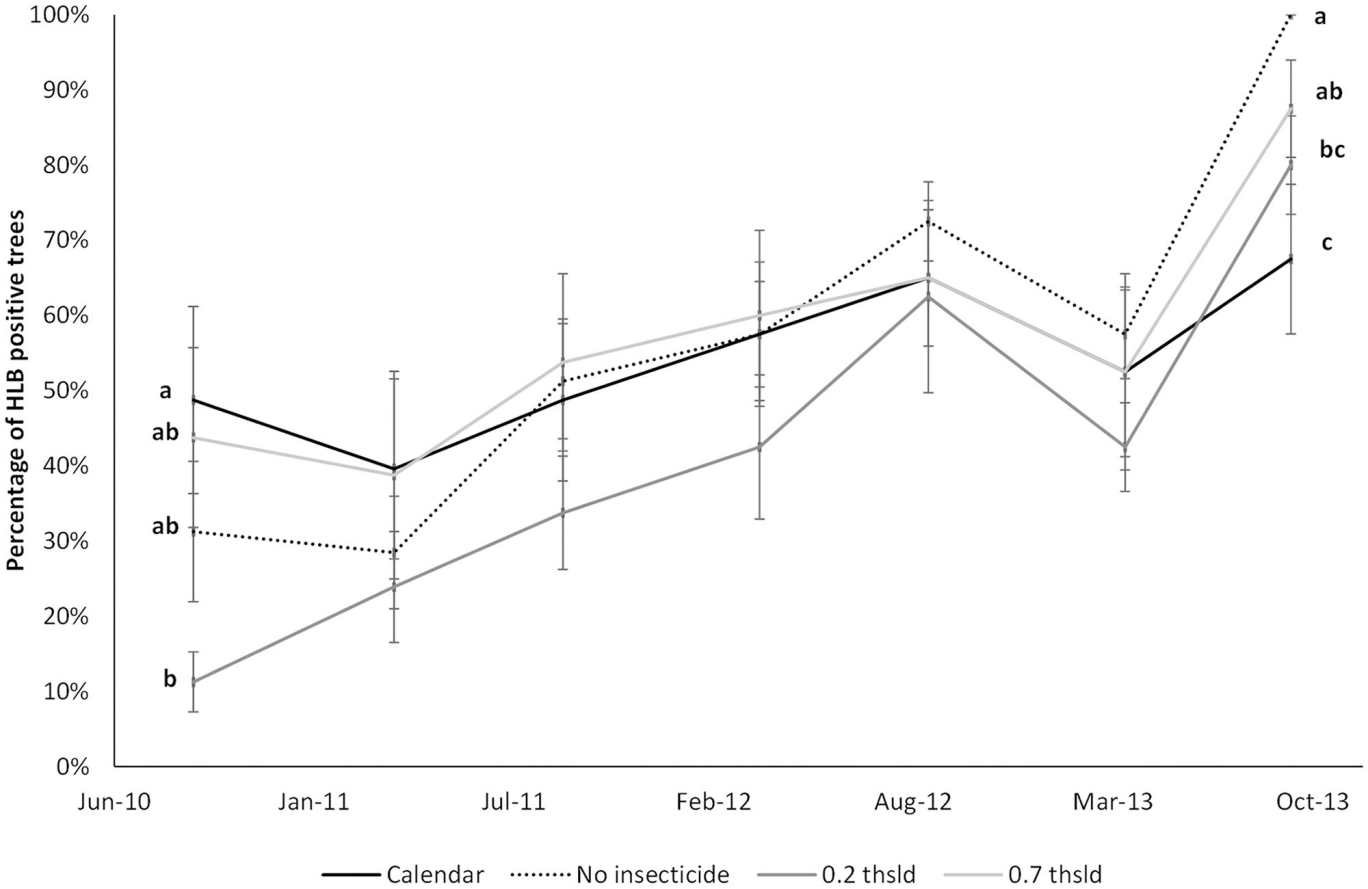
Percentage (mean ± SE) of trees at Site 2 testing positive by qPCR analysis. DNA was extracted from petiole tissue samples collected at the beginning of the experiment (September 2010) and at two sampling dates during 2011, 2012, and 2013, in plots under four different vector management strategies: monthly calendar insecticide applications ‘calendar’, insecticide applications based on a nominal threshold of 0.2 adults per stem-tap ‘0.2 thsld’, applications based on a nominal threshold of 0.7 adults per stem-tap ‘0.7 thsld’, and no insecticide applications to control ACP ‘no insecticide’.

At Site 1, 1,117 of the 1,120 samples analyzed by qPCR throughout the study were positive for *C*. L. asiaticus indicating that practically 100% of the trees on this study site were HLB infected from the onset of the experiment. In Site 2, HLB incidence ranged between 10 and 50% of trees testing positive at the beginning of the experiment with differences between treatments (F = 9.50; df = 3, 25; *P* = 0.0004) associated with uncontrolled variability in the grove such as edge effects. Incidence increased with time with no significant treatment effects until the last sampling date (March 2011: F = 1.97; df = 3, 25; *P* = 0.1436; September 2011: F = 2.71; df = 3, 25; *P* = 0.0668; March 2012: F = 1.03; df = 3, 25; *P* = 0.3968; September 2012: F = 0.35; df = 3, 25; *P* = 0.7918; March 2013: F = 0.65; df = 3, 25; *P* = 0.5899; September 2013: F = 3.49; df = 3, 25; *P* = 0.0304, Fig 2). At the end of the experiment, all sampled trees in the ‘no insecticide’ treatment tested positive for HLB compared to 88.5 ± 6.4% in trees subjected to the ‘0.7 thsld’, 80.0 ± 6.5% to the ‘0.2 thsld’ and 67.5 ± 10.0% for the calendar treatment, the latter being significantly less than either the ‘no insecticide’ or ‘0.7 thsld’ treatments.

### Harvest evaluations

No treatment effects were observed on juice per kg fruit, solids per fruit or acidity for any of the harvests in Site 1 (Table 5). However, the brix:acid ratio was lower in fruit harvested in 2011 and brix was higher in 2013 in fruit from trees receiving calendar sprays compared to untreated or ‘0.2 thsld’ treatments and untreated and ‘0.7 thsld’ respectively, although no such effects were observed at any other harvest (Table 5). At Site 2, significantly less juice per kg fruit was obtained from ‘calendar’ sprayed trees compared to the remaining treatments harvested in 2012. Similarly, soluble solids were significantly less in fruit from ‘calendar’ plots compared to the rest in 2011 and 2012 and for acid in 2012. No significant treatment effects were found at the last (2014) harvest nor for total degrees brix and brix/acid ratio parameters from any of the four harvests.

**Table 5:** Kilograms of juice per kilogram of fruit, kilogram solids per kilogram of fruit, acidity, degrees brix, and brix/acid ratio (mean ± SE) in juice from fruit samples of the four treatments evaluated in two citrus commercial groves: Monthly calendar insecticide applications ‘calendar’, insecticide applications based on a nominal threshold of 0.2 adults per stem tap ‘0.2 thsld’, applications based on a nominal threshold of 0.7 adults per stem tap ‘0.7 thsld’, and no insecticide applications to control ACP ‘no insecticide’. Harvest of the ‘Earlygold’ variety occurred during the first year of the season (Site 1) and of ‘Valencia’ in the second year of the season (Site 2). Values marked in bold indicate significant treatment effects. Means for treatments followed by the same letter for a particular parameter and within a particular year are not significantly different (LSD Means: P <0.05).

			kg juice per kg fruit	kg solids per kg fruit	Acid	Total Brix	Ratio
**Site 1**	**2010–11**	**No insecticide**	0.557 ± 0.009	0.0711 ± 0.0012	0.505 ± 0.006	12.63 ± 0.3	25.04 ± 0.84
		**0.7 thsld**	0.535 ± 0.012	0.0682 ± 0.002	0.46 ± 0.01	12.77 ± 0.29	27.8 ± 0.96
		**0.2 thsld**	0.547 ± 0.007	0.0692 ± 0.0011	0.475 ± 0.024	12.67 ± 0.16	26.82 ± 1.02
		**Calendar**	0.561 ± 0.016	0.0686 ± 0.0021	0.5 ± 0.015	12.26 ± 0.42	24.57 ± 1.05
			*(F = 0*.*92; df = 3*, *9; P = 0*.*4709)*	*(F = 0*.*56; df = 3*, *9; P = 0*.*6522)*	*(F = 1*.*92; df = 3*, *9; P = 0*.*1973)*	*(F = 0*.*87; df = 3*, *9; P = 0*.*4933)*	*(F = 1*.*93; df = 3*, *9; P = 0*.*1955)*
	**2011–12**	**No insecticide**	0.525 ± 0.014	0.0638 ± 0.0018	0.403 ± 0.006	12.16 ± 0.11	**30.22 ± 0.47b**
		**0.7 thsld**	0.558 ± 0.02	0.0682 ± 0.0025	0.378 ± 0.01	12.22 ± 0.05	**32.45 ± 0.91ab**
		**0.2 thsld**	0.592 ± 0.025	0.0717 ± 0.0028	0.403 ± 0.017	12.12 ± 0.06	**30.25 ± 1.09b**
		**Calendar**	0.574 ± 0.019	0.0701 ± 0.0024	0.368 ± 0.005	12.22 ± 0.08	**33.26 ± 0.51a**
			*(F = 1*.*93; df = 3*, *9; P = 0*,*1946)*	*(F = 1*.*69; df = 3*, *9; P = 0*.*238)*	*(F = 3*.*12; df = 3*, *9; P = 0*.*0806)*	*(F = 0*.*44; df = 3*, *9; P = 0*.*7295)*	***(F = 4*.*08; df = 3*, *9; P = 0*.*0438)***
	**2012–13**	**No insecticide**	0.56 ± 0.011	0.0652 ± 0.0031	0.39 ± 0.007	11.62 ± 0.37	29.87 ± 1.38
		**0.7 thsld**	0.568 ± 0.026	0.0661 ± 0.0028	0.405 ± 0.023	11.65 ± 0.26	29.11 ± 2.03
		**0.2 thsld**	0.553 ± 0.009	0.0658 ± 0.0027	0.407 ± 0.013	11.9 ± 0.4	29.34 ± 1.58
		**Calendar**	0.547 ± 0.016	0.0624 ± 0.0011	0.412 ± 0.05	11.42 ± 0.2	28.75 ± 3.06
			*(F = 0*.*24; df = 3*, *9; P = 0*.*8647)*	*(F = 0*.*59; df = 3*, *9; P = 0*.*6366)*	*(F = 0*.*12; df = 3*, *9; P = 0*.*9467)*	*(F = 1*.*04; df = 3*, *9; P = 0*.*4207)*	*(F = 0*.*06; df = 3*, *9; P = 0*.*9804)*
	**2013–14**	**No insecticide**	0.572 ± 0.016	0.0728 ± 0.0019	0.331 ± 0.007	**12.72 ± 0.12a**	38.54 ± 0.97
		**0.7 thsld**	0.588 ± 0.014	0.0749 ± 0.0018	0.348 ± 0.007	**12.74 ± 0.16a**	36.64 ± 0.91
		**0.2 thsld**	0.58 ± 0.01	0.072 ± 0.0017	0.326 ± 0.01	**12.41 ± 0.12ab**	38.29 ± 1.34
		**Calendar**	0.591 ± 0.006	0.072 ± 0.0016	0.32 ± 0.008	**12.19 ± 0.19b**	38.2 ± 0.88
			*(F = 0*.*24; df = 3*, *25; P = 0*.*8647)*	*(F = 0*.*64; df = 3*, *25; P = 0*.*5983)*	*(F = 2*.*17; df = 3*, *25; P = 0*.*1167)*	***(F = 3*.*29; df = 3*, *25; P = 0*.*0371)***	*(F = 0*.*64; df = 3*,*25; P = 0*.*5983)*
**Site 2**	**2010–11**	**No insecticide**	0.474 ± 0.01	0.0573 ± 0.001	1.19 ± 0.017	12.08 ± 0.12	10.17 ± 0.18
		**0.7 thsld**	0.496 ± 0.024	0.0576 ± 0.0012	1.248 ± 0.029	12.24 ± 0.23	9.82 ± 0.12
		**0.2 thsld**	0.493 ± 0.016	0.0583 ± 0.0015	1.241 ± 0.035	11.87 ± 0.28	9.58 ± 0.14
		**Calendar**	0.482 ± 0.004	0.0577 ± 0.0011	1.194 ± 0.043	11.97 ± 0.22	10.08 ± 0.23
			*(F = 0*.*41; df = 3*, *25; P = 0*.*7463)*	(F = 0.14; df = 3, 25; P = 0.935)	*(F = 0*.*88; df = 3*, *25; P = 0*.*4625)*	*(F = 0*.*58; df = 3*, *25; P = 0*.*6344)*	*(F = 2*.*31; df = 3*, *25; P = 0*.*1002)*
	**2011–12**	**No insecticide**	**0.553 ± 0.006a**	**0.0701 ± 0.0006a**	0.853 ± 0.024	12.68 ± 0.07	14.91 ± 0.41
		**0.7 thsld**	**0.557 ± 0.012a**	**0.0717 ± 0.0024a**	0.915 ± 0.023	12.86 ± 0.18	14.08 ± 0.29
		**0.2 thsld**	**0.55 ± 0.013a**	**0.0714 ± 0.0024a**	0.873 ± 0.032	12.97 ± 0.15	14.91 ± 0.48
		**Calendar**	**0.525 ± 0.008b**	**0.0658 ± 0.0007b**	0.818 ± 0.013	12.54 ± 0.07	15.34 ± 0.17
			*(F = 9*.*53; df = 3*, *9; P = 0*.*0037)*	***(F = 60*.*02; df = 3*, *9; P = 0*.*0155)***	*(F = 3*.*5; df = 3*, *9; P = 0*.*0626)*	*(F = 2*.*22; df = 3*, *9; P = 0*.*1557)*	*(F = 2*.*61; df = 3*, *9; P = 0*.*1158)*
	**2012–13**	**No insecticide**	0.618 ± 0.01	**0.0776 ± 0.0012a**	**0.813 ± 0.016a**	12.56 ± 0.08	15.5 ± 0.34
		**0.7 thsld**	0.598 ± 0.007	**0.0761 ± 0.0015a**	**0.818 ± 0.016a**	12.72 ± 0.15	15.6 ± 0.36
		**0.2 thsld**	0.601 ± 0.012	**0.0766 ± 0.0016a**	**0.806 ± 0.011a**	12.75 ± 0.08	15.83 ± 0.21
		**Calendar**	0.584 ± 0.01	**0.0717 ± 0.0019b**	**0.74 ± 0.014b**	12.27 ± 0.18	16.63 ± 0.43
			*(F = 2*.*39; df = 3*, *25; P = 0*.*0928)*	***(F = 3*.*21; df = 3*, *25; P = 0*.*04****)*	***(F = 6*.*66; df = 3*, *25; P = 0*.*0018)***	*(F = 2*.*55; df = 3*, *25; P = 0*.*0781)*	*(F = 2*.*11; df = 3*, *25; P = 0*.*1248)*
	**2013–14**	**No insecticide**	0.58 ± 0.015	0.0724 ± 0.0028	0.669 ± 0.028	12.45 ± 0.2	18.91 ± 1.01
		**0.7 thsld**	0.59 ± 0.003	0.076 ± 0.0013	0.716 ± 0.023	12.9 ± 0.25	18.08 ± 0.43
		**0.2 thsld**	0.574 ± 0.01	0.0745 ± 0.002	0.705 ± 0.028	12.96 ± 0.14	18.68 ± 1.05
		**Calendar**	0.588 ± 0.003	0.0751 ± 0.0014	0.656 ± 0.026	12.76 ± 0.23	19.67 ± 0.91
			*(F = 0*.*57; df = 3*, *25; P = 0*.*6389)*	*(F = 0*.*6; df = 3*, *25; P = 0*.*6208)*	*(F = 1*.*16; df = 3*, *25; P = 0*.*3439)*	*(F = 1*.*24; df = 3*, *25; P = 0*.*3169)*	*(F = 0*.*51; df = 3*, *25; P = 0*.*677)*

Significant effects on yield (kg solids per ha) were found in Site 1 (‘Earlygold’) for all but the first (2010) harvest and over all 4 harvests (F = 4.27; df = 3, 12.85; *P* = 0.0267, Table 6). ‘Calendar’ treatments resulted in greatest yields at all of the 3 last harvests, although not significantly different from the control or ‘0.7 thsld’ in 2012–13. Time effects were significant (F = 11.68; df = 3, 36.36; *P* < 0.0001) but some varied among treatments (treatment x time interaction: F = 2.00; df = 9, 36.70; *P* = 0.067). Yields from trees sprayed by the ‘calendar’ or 0.2 threshold increased over the 4 year study (‘calendar’: t-value = -4.44; df = 44.32; P < 0.0001; ‘0.2 thsld’: t-value = -3.63; df = 44.32; *P* = 0.0007), but marginally from unsprayed trees (t-value = -1.92; df = 44.32; *P* = 0.061). No yield increases were observed in trees sprayed on a 0.7 threshold (t-value = -1.32; df = 44.32; *P* = 0.19).

**Table 6:** Absolute yields expressed as kg of soluble solids per hectare (mean ± SE) in each of the four treatments tested (monthly calendar insecticide applications ‘calendar’, insecticide applications based on a nominal threshold of 0.2 adults per stem tap ‘0.2 thsld’, applications based on a nominal threshold of 0.7 adults per stem tap ‘0.7 thsld’, and no insecticide applications to control ACP ‘no insecticide’) in two commercial citrus groves during 2010–11, 2011–12, 2012–13 and 2013–14 seasons. Harvest of the ‘Earlygold’ variety occurred during the first year of the season (Site 1) and of ‘Valencia’ in the second year of the season (Site 2). Values marked in bold indicate significant treatment effects for that particular season and grove. Mean yields for treatments followed by the same letter within a particular year are not significantly different (LSD Means: P <0.05).

Season					
		2010–11	2011–12	2012–13	2013–14
**Site 1**	**No spray**	1493.51 ± 87.61	**1433.46 ± 87.54b**	**1560.05 ± 132.68ab**	**1709.76 ± 33.7b**
	**0.7 thsld**	1468.84 ± 108.2	**1523.86 ± 102.56b**	**1615.49 ± 130.82ab**	**1617.61 ± 71.45b**
	**0.2 thsld**	1370.89 ± 97.82	**1463.86 ± 173.66b**	**1485.76 ± 205.16b**	**1779.44 ± 120.06b**
	**Calendar**	1515.78 ± 60.09	**1816.93 ± 56.9a**	**1727.39 ± 81.6a**	**2014.54 ± 51.63a**
**Site 2**	**No spray**	1703.2 ± 234.46	1670.55 ± 56.52	**1887.77 ± 113.51bc**	**1281.89 ± 73.33b**
	**0.7 thsld**	1512.69 ± 198.03	1711.75 ± 314.24	**1765.26 ± 270.1c**	**1527.17 ± 258.82b**
	**0.2 thsld**	1239.82 ± 309.39	1873.94 ± 446.44	**2142.54 ± 152.99ab**	**1856.86 ± 245.44a**
	**Calendar**	1603.36 ± 376.39	1812.68 ± 385.03	**2289.94 ± 141.2a**	**1962.02 ± 152.46a**

Treatment effects in Site 2 (‘Valencia’) were significant for the last two harvests. Again, highest yields were seen from the ‘calendar’ treatment although not significantly different from ‘02 thsld’ (Table 6). Although no treatment effects were observed over all 4 harvests (F = 0.41; df = 3, 45; *P* = 0.7472), the treatment x time interaction was significant (F = 2.50; df = 9, 45; *P* = 0.0205) with a general trend toward increasing yields from both high input treatments for the 2012 and 2013 harvests (‘calendar’: t-value = -2.39; df = 45.00; *P* = 0.0211, ‘0.2 thsld’: t-value = -3.84; df = 45.00; *P* = 0.0004) that was not observed with the two low input treatments (‘0.7 thsld’: t-value = -0.78; df = 45.00; *P* = 0.4394, ‘no insecticide’: t-value = -0.69; df = 45.00; *P* = 0.4923). However, the trend varied at the last (2014) harvest when a sharp increase in ACP densities that season coincided with reduced yields from all treatments with respect to the previous season (‘calendar’: t-value 2.78; df = 45.00; *P* = 0.0079, ‘0.2 thsld’: t-value = 2.80; df = 45.00; *P* = 0.0075, ‘0.7 thsld’: t-value = 2.67; df = 45.00; *P* = 0.0106, ‘no insecticide’: t-value = 6.70; df = 45.00; *P* < 0.001). Yield reduction was most notable in ‘no insecticide’ plots (Table 6).

### Economic study

‘Calendar’ ACP insecticide management increased grove caretaking costs over the untreated by approximately $1,500 per ha and season. Treatment costs based on nominal thresholds were lower than calendar sprays, but varied from year to year depending on ACP populations (Table 4). Delivered-in break-even prices covering all production costs were highest ($5.14 per kg solids) and lowest ($3.89 per kg solids) in untreated plots, for the 2011–2012 and 2013–2014 seasons respectively. This contrasted with results from Site 2 where extremes of $5.22 and $3.66 the 2010–2011 and 2013–2014 harvests resulted instead from the 0.2 threshold treatment (Table 7). When costs and revenues from the last three harvests were summed, break-even prices were lowest with the ‘0.7 thsld’ ($4.42) and highest with the ‘0.2 thsld’ ($4.56) treatments in Site 1. Opposite results were obtained at Site 2: highest with the ‘0.7 thsld’ ($4.13) and lowest with the ‘0.2 thsld’ ($3.79).

**Table 7:** Profitability of the citrus operation subjected to the four treatments tested (ACP control through calendar insecticide applications ‘calendar’, ACP management based on a 0.2 ACP per stem-tap nominal threshold ‘0.2 thsld’, ACP management based on a 0.7 threshold ‘0.7 thsld’, no insecticide ACP control ‘no insecticide’) in two commercial groves at three price scenarios (low: 3.79, medium: 5.24, high: 5.38 $ per kg of solids) for 4 seasons between 2011 and 2015 and for the sum of the last three seasons. Bold positive values indicate that the grower would recover the total cost of production.

			Solids (kg/ha)	Revenues ($/ha)			Total costs ($/ha)	Delivered-in ($/kg solid)	Profitability ($/ha)		
				3.79	5.24	5.38			3.79	5.24	5.38
**Site 1**	2010–11*	**No insecticide**	1532	5806	8027	8242	6554	4.28	-748	**1473**	**1687**
		**0.7 thsld**	1446	5482	7579	7782	6523	4.51	-1041	**1056**	**1259**
		**0.2 thsld**	1369	5190	7176	7368	6399	4.67	-1209	**777**	**968**
		**Calendar**	1502	5691	7868	8078	6582	4.38	-892	**1286**	**1496**
	**2011–12**	**No insecticide**	1336	5063	7000	7187	6860	5.14	-1797	**140**	**327**
		**0.7 thsld**	1518	5754	7955	8168	7201	4.74	-1447	**755**	**967**
		**0.2 thsld**	1534	5812	8036	8251	7124	4.65	-1312	**912**	**1127**
		**Calendar**	1861	7052	9750	10011	8428	4.53	-1375	**1323**	**1583**
	**2012–13**	**No insecticide**	1568	5942	8215	8435	7111	4.54	-1170	**1104**	**1323**
		**0.7 thsld**	1646	6240	8627	8857	7398	4.49	-1158	**1229**	**1459**
		**0.2 thsld**	1507	5713	7899	8110	7306	4.85	-1593	**592**	**804**
		**Calendar**	1661	6294	8702	8935	8439	5.08	-2145	**263**	**496**
	**2013–14**	**No insecticide**	1706	6467	8941	9180	6636	3.89	-169	**2305**	**2544**
		**0.7 thsld**	1661	6295	8703	8936	6749	4.06	-454	**1955**	**2187**
		**0.2 thsld**	1757	6659	9206	9452	7458	4.24	-799	**1748**	**1994**
		**Calendar**	1990	7543	10429	10707	8158	4.10	-615	**2271**	**2550**
	**Cumulative**	**No insecticide**	4610	17472	24156	24801	20607	4.47	-3136	**3549**	**4194**
		**0.7 thsld**	4826	18289	25286	25961	21348	4.42	-3059	**3938**	**4614**
		**0.2 thsld**	4798	18184	25141	25813	21888	4.56	-3704	**3253**	**3925**
		**Calendar**	5512	20889	28881	29653	25024	4.54	-4135	**3857**	**4629**
**Site 2**	2010–11*	**No insecticide**	1690	6404	8854	9091	7251	4.29	-847	**1604**	**1840**
		**0.7 thsld**	1509	5720	7908	8119	6962	4.61	-1242	**946**	**1157**
		**0.2 thsld**	1253	4750	6567	6743	6548	5.22	-1798	**19**	**195**
		**Calendar**	1602	6073	8397	8621	7099	4.43	-1026	**1298**	**1522**
	**2011–12**	**No insecticide**	1648	6247	8637	8868	6641	4.03	-394	**1996**	**2227**
		**0.7 thsld**	1726	6543	9046	9288	6904	4.00	-361	**2142**	**2384**
		**0.2 thsld**	1883	7136	9866	10130	7186	3.82	-50	**2680**	**2943**
		**Calendar**	1813	6870	9498	9752	8257	4.55	-1387	**1242**	**1495**
	**2012–13**	**No insecticide**	1909	7234	10001	10269	7128	3.73	106	**2874**	**3141**
		**0.7 thsld**	1750	6633	9171	9416	7248	4.14	-615	**1923**	**2168**
		**0.2 thsld**	2137	8100	11199	11498	7822	3.66	**278**	**3377**	**3677**
		**Calendar**	2290	8679	11999	12320	8733	3.81	-54	**3267**	**3587**
	**2013–14**	**No insecticide**	1245	4720	6526	6700	6024	4.84	-1304	**502**	**676**
		**0.7 thsld**	1559	5907	8167	8385	6622	4.25	-715	**1545**	**1764**
		**0.2 thsld**	1857	7039	9732	9992	7245	3.90	-206	**2487**	**2747**
		**Calendar**	1977	7493	10360	10637	7922	4.01	-429	**2438**	**2714**
	**Cumulative**	**No insecticide**	4802	18201	25165	25837	19793	4.12	-1592	**5372**	**6044**
		**0.7 thsld**	5035	19083	26384	27089	20773	4.13	-1690	**5611**	**6316**
		**0.2 thsld**	5877	22275	30797	31620	22253	3.79	**22**	**8544**	**9367**
		**Calendar**	6080	23042	31858	32709	24912	4.10	-1870	**6946**	**7797**

* Management costs during the first season were considered as the average ones estimated for southwest Florida by Muraro and Roka (2013).

At the low ($3.79/kg solids) price, none of the 4 management strategies would have been profitable for any season in either site except the ‘0.2 thsld’ treatment at Site 2 for the 2013–2014 season and for the sum of the last three seasons. In contrast, all treatments were profitable in all seasons at the high $5.24 per kg solids scenario (Table 7). In general, strategies incurring higher ACP management costs became relatively more profitable as juice prices increased, although profitability varied among groves, treatments and seasons. For example, the ‘0.2 thsld’ strategy resulted most profitable at Site 2 for all but the first season and over all dates but never at Site 1.

### Economic injury level model

**Fig 3:**
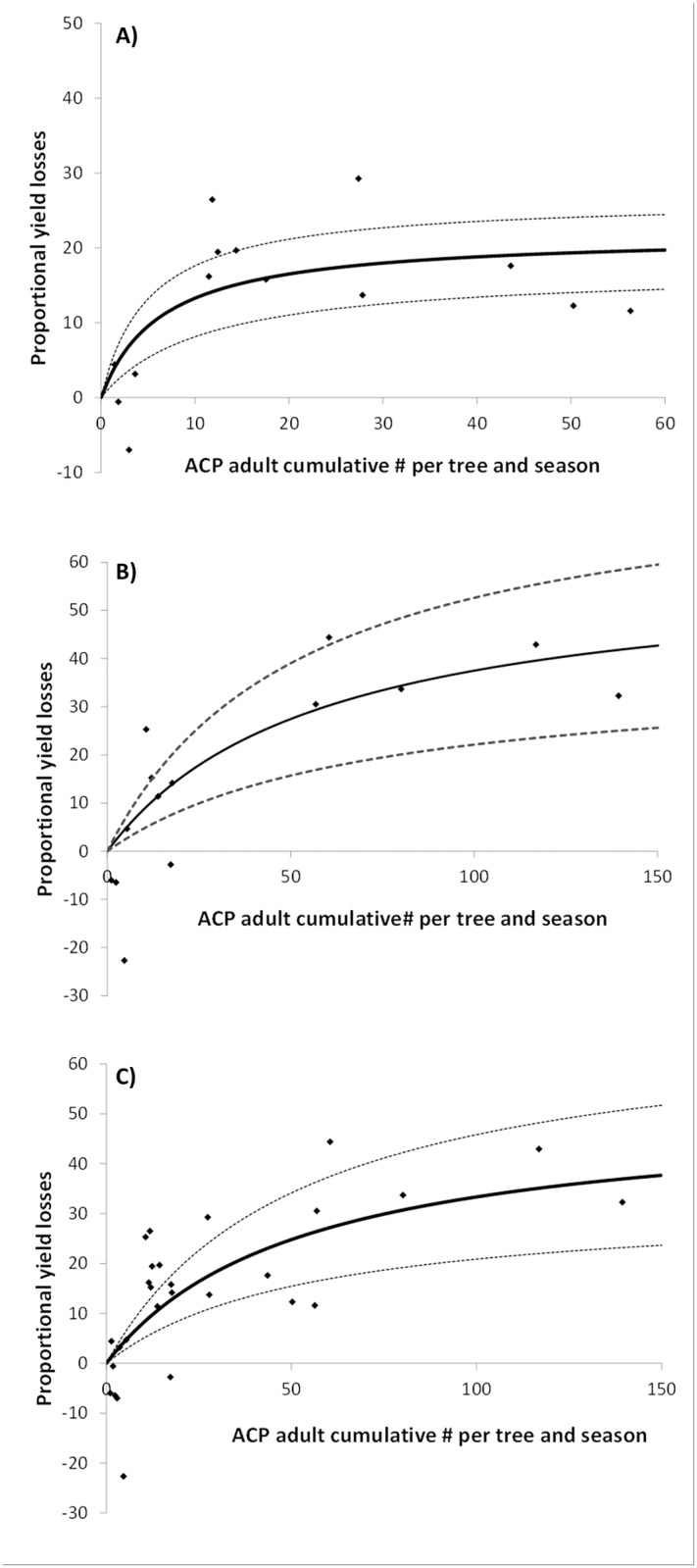
Rectangular hyperbolic equations. Equations were estimated by non-linear regression relating proportional yield losses during the 2013–14 season harvest of all plots with respect to ‘calendar’ plots with ACP cumulative numbers per stem-tap, tree and season after three and half years of study: **A)** Site 1, **B)** Site 2 and **C)** the two groves pooled. Dotted lines represent maximum and minimum values of *ρ* for each *κ* value using the maximum and minimum estimated values of the parameters ‘A’ and ‘I’ given by their estimated standard errors.

Average yields in plots receiving monthly calendar insecticide applications (*Y*_*m*_) were 2,014.5 ± 51.6, 1,962.0 ± 245.4 and 1,988.3 ± 75.2 kg of soluble solids per ha in Site 1, Site 2, and pooled over the two groves respectively. Using the ‘calendar’ plots (*Y*_*m*_) as a baseline, the rectangular hyperbolic model indicated a significant relationship between yield losses (*ρ*) in each plot on the one hand, and ACP adult cumulative numbers per stem-tap and tree (*κ*) on the other, for either site alone and pooled (Site 1: F = 25.00; df = 2, 14; *P* < 0.0001; Pseudo-R^2^ = 0.46; Site 2: F = 24.48; df = 2, 14; *P* < 0.0001; Pseudo-R^2^ = 0.68; two sites pooled: F = 38.10; df = 2, 30; *P* < 0.0001; Pseudo-R^2^ = 0.52) (Fig 3). Estimated slopes at the origin were I = 3.39 ± 1.85 for Site 1, I = 1.03 ± 0.49 for Site 2 and I = 0.97 ± 0.38 for the two groves pooled. The estimated horizontal asymptotes that define maximum yield losses (%) were A = 21.80 ± 4.68 for Site 1, A = 59.06 ± 21.61 for Site 2 and A = 50.91 ± 18.69 for the two groves pooled. Substituting these values for ‘*Y*_*m*_’, ‘I’ and ‘A’ values in Eq (4) provided the following equations for Site 1 Eq (5.1), Site 2 Eq (5.2) and the two groves pooled Eq (5.3):

$$C=P\cdot 2014.5\cdot \left(\frac{3.39\cdot k}{1+\frac{3.39\cdot k}{21.8}}\right)\cdot 100^{-1}$$ (5.1)

$$C=P\cdot 1962\cdot \left(\frac{1.03\cdot k}{1+\frac{1.03\cdot k}{59.06}}\right)\cdot 100^{-1}$$ (5.2)

$$C=P\cdot 1988.3\cdot \left(\frac{0.97\cdot k}{1+\frac{0.97\cdot k}{50.91}}\right)\cdot 100^{-1}$$ (5.3)

Given known ACP management costs (*C*) and juice prices (*P*), the EIL could thus be defined by the cumulative number of ACP adults per stem-tap, tree and season (*κ*) that balanced the two sides of these equations [S1 File].

### EIL implementation

The cumulative number of adults per stem-tap, tree and season over which it would be more cost-effective to spray than not to spray is calculated from Eq (5) considering an orange juice price of $5.25 per kg of solids ($2.38 per pound-solids), approximating spraying costs at $75/ha and $130/ha for dormant and growing season respectively, and allocating $141 per ha and season for ACP monitoring **(**Table 8**)**.

**Table 8:** Estimated economic injury levels expressed as the cumulative number of ACP adults obtained per stem-tap and tree during one season (Mean [lower and upper Standard Error Intervals]) above which it would be more cost-effective to spray than not to spray based on application costs incurred during this study. Juice price variable was fixed on $5.25 per kg of solids. Estimations were made for the EIL models obtained in Site 1 (100% HLB incidence), Site 2 (83% incidence at the end of the experiment) and the two groves pooled. Dormant-season insecticide total costs per application were estimated at $75 per ha, growing season total costs per application at $130 per ha, and monitoring costs at $141 acre.

	ACP management costs ($/ha)	Cumulative ACP per tap and season		
Number of applications		Site 1	Site 2	Two groves pooled
2 dormant + 1 growing-season	421	1.4 [0.9–3.4]	4.3 [2.9–8.5]	4.5 [3.2–7.8]
2 dormant + 2 growing-season	551	2.0 [1.2–4.9]	5.7 [3.8–11.5]	6.1 [4.2–10.7]
2 dormant + 3 growing-season	681	2.7 [1.6–6.7]	7.3 [4.8–14.9]	7.7 [5.4–13.9]
2 dormant + 4 growing-season	811	3.5 [9.0–2.1]	8.9 [5.8–18.4]	9.5 [6.5–17.4]
2 dormant + 5 growing-season	941	4.4 [2.6–12]	10.5 [22.4–6.8]	11.3 [7.7–21.3]
2 dormant + 6 growing-season	1071	5.6 [3.1–16.1]	12.3 [7.9–26.6]	13.3 [9.0–25.6]
2 dormant + 7 growing-season	1201	7.0 [3.8–21.9]	14.2 [9.1–31.3]	15.4 [10.3–30.4]

## Discussion

Insecticide applications reduced ACP populations as expected, commensurate with frequency of sprays but variably depending on season and location. Ecological factors such as environmental and community variables or specific landscape structure may modulate ACP pressure on citrus groves [41, 42]. We found ACP densities to rarely reach the two pre-established nominal thresholds (0.2 and 0.7 ACP adults per stem-tap) from 2010 to 2012. Consequently, few applications were needed to maintain ACP populations below threshold levels. More applications were needed to hold adults below the 0.2 adults per tap threshold during the last two seasons, especially at Site 2. The variable number of applications needed to maintain thresholds among years translated into varying costs and thus profitability of the prefixed nominal threshold management approach (Table 7). Other factors such as insecticide or fuel costs and juice prices provide additional uncertainty that counterbalances, to some degree, the potential advantage of pre-fixed nominal threshold based decision making.

HLB was near 100% from the outset in Site 1 but progressed in Site 2 from a lower initial point over the four seasons of data collection. Given the current epidemiological model of transmission within a single ACP generation [41] it is likely that trees testing positive for the first time during the early years of the experiment had been infected for some time. While HLB progressed more rapidly in plots without insecticide management toward the end, even monthly sprays resulting in near zero stem-tap numbers were insufficient to halt the epidemic. Belasque *et al*. [16] reported that even more frequent sprays, (usually between 13 and 21 per season), plus the elimination of infected trees, effectively reduced HLB expansion. However, HLB incidence was lower than in our study and blocks and more than 28% HLB infected trees were completely eliminated.

Yield response to vector control was observed at Site 1 despite almost 100% of the trees being already PCR positive at the beginning of the study. Direct damage produced by ACP and potential damage attributed to other citrus pests in plots not under ACP insecticidal control might, in part, explain some yield differences between treatments. However, ACP direct damage at the pest densities observed in this study is considered negligible and potential damage caused by other citrus pests was minimized by targeted treatment before populations attained levels that may have risked production.

All this suggests that suppression of the vector population reduced HLB intensity. Although our data did not show a relationship between titer and treatment, a follow-up study from fruit samples collected in our plots over 3 years and using a more sensitive PCR protocol did show significantly lower *C*Las titer in juice from trees under insecticidal control [14]. This is the best direct evidence we have so far for a direct relationship between bacterial titer and vector control in already HLB infected trees. Beneficial effects of vector control to infected trees could occur through curtailed reinoculation of bacteria reducing the number of infections per tree [12]. Furthermore, absence of vectors could allow new flush to develop uninfected, perhaps improving flow of photosynthate to starved roots, reversing degeneration and improving ability to absorb and translocate soil nutrients [43]. In this study, we saw that yield increased with frequency of sprays and consequent decline of ACP populations. These results provide further evidence that HLB-infected trees can remain productive, at least in the medium term, through efficient vector control together with horticultural practices that further relieve stress on infected trees [5, 6].

We did not find noticeable treatment effects on juice quality measures. Statistical differences appeared sporadically without a general pattern throughout the study. This ran contrary to the widespread belief that HLB affects juice quality. Bitter flavors associated to HLB and attributed to increased concentrations of limonin and nomilin rates [44] were not evaluated in the present study. However, juice market prices are solely based on the measured amount of soluble solids which did not respond positively to vector control in this or previous studies [5, 6]. On the contrary, we found lower solids at some harvests from trees under more intensive insecticidal control. However, no general pattern emerged, and the measured amount of soluble solids from both groves was higher than the recent local average of 0.069 kg of solids per kg of fruit [36].

Under conditions of this study, juice prices above $4 per kg of soluble solids were necessary to maintain profitability. Fortunately such prices have been the norm in recent years [9]. High break-even prices are mostly due to sharp increments in management costs, much of which have been devoted to ACP control. In our study, foliar nutrition constituted approximately 9% of total costs compared to 14% for insecticides to control ACP with calendar sprays. Nominal threshold treatments reduced these latter by more than half, although costs were variable depending on season and location. On the other hand, material costs of nutrients and insecticides quoted to us by retailers were probably higher than many growers are able to negotiate [36]. Furthermore, we chose insecticides based on a resistance management and natural enemy conservation approach of using selective insecticides during the growing season and not repeating modes of action rather than economizing product choices. Taking these factors into account, even juice prices somewhat below $4 per kg of solids could be marginally profitable, at least in the medium term, by reducing input costs. Nevertheless, the relatively narrow profit margins under our study conditions underline the importance of developing more efficient vector control strategies.

We were able to mathematically relate yield with ACP densities measured as adults per stem-tap. The hyperbolic model suggested somewhat different results between Site 1, with high HLB incidence but relatively low ACP density, compared to Site 2 with initially moderate HLB incidence but higher ACP population. We also saw reduced incidence of HLB with calendar sprays toward the end of the study at Site 2, which undoubtedly provided an additional benefit to those trees. This result underscores the difference between protecting trees from initial infection versus from re-inoculation.

Our proposed ACP insecticide management approach for cases of high HLB incidence when cost variables are known can optimize the frequency of insecticide applications according to the specific circumstances of each season (example: Table 8). Decreasing spray costs would decrease corresponding thresholds accordingly. The number of insecticide applications and marginal profits would also vary depending on pest pressure and efficacy ACP control. The estimated dynamic treatment levels thus overcome the variability of economical profits inherent in pre-fixed nominal thresholds.

Threshold-based management as we define it here is limited to HLB infected mature trees and thus does not take into account risk of HLB infection to newly planted trees. The eventual loss of production in response to ACP populations on young trees with little or no incidence of HLB has yet to be evaluated, but such a threshold would undoubtedly be low. Young trees have greater potential value than older trees, are more susceptible to HLB and flush more frequently, so are highly attractive to ACP. On the other hand, soil applied systemic insecticides and UV reflective mulch are effective measures for controlling ACP in new plantings in addition to foliar sprays [1, 45]. Therefore, there is potential to integrate a threshold approach with area-wide management such as has been implemented in Florida that already includes an intensive monitoring program providing stem-tap data at 3-week intervals from a significant portion of the citrus acreage [www.flchma.org]. Controlling spray frequency in this way could open the door for additional management tactics, including better biological control through conservation of natural enemies. While several studies have demonstrated that ACP mortality attributed to predators or parasitoids does not provide sufficient control of ACP populations under present circumstances, natural enemy assemblages can significantly reduce ACP numbers during the growing season [18, 25, 26]. Better regulation of ACP populations by natural enemies would directly translate into less frequent insecticide applications and consequently higher profits.

Finally, although our studies were conducted in two large commercial citrus blocks, the economic injury level models obtained for vector control under moderate-to-high HLB pressure should be validated on a larger scale to account for effects of location or specific management operations. Furthermore, integration of effective young tree programs would be necessary to apply threshold based management over a wide area that would naturally include citrus of all ages and levels of HLB incidence.

## Supporting information

S1 File Economic injury level estimator. Estimator for the ACP cumulative number (cell **I17** of the suporting information file) that balances ACP insecticide managament costs with yield losses associated to this pests for variable juice prices (this variable can be defined at cell **I11** of the suporting information file) and insecticide management costs (this variable can be defined at cell **H3** of the suporting information file. Estimations are done using Eq (5) for Sites 1 and 2 and for the data of the two groves pooled.
(XLSX)
